# Dr. John David Davis

**Published:** 1912-05

**Authors:** 


Dr. John David Davis of Springville, died March 26, 1912, aged 52. He was a graduate of the University of Buffalo, 1886. He had resided at Westfield before going to Springville.

